# Association between plasma adipsin level and mild cognitive impairment in Chinese patients with type 2 diabetes: a cross-sectional study

**DOI:** 10.1186/s12902-019-0431-y

**Published:** 2019-10-24

**Authors:** Dan Guo, Yang Yuan, Rong Huang, Sai Tian, Jiaqi Wang, Hongyan Lin, Ke An, Jin Han, Shaohua Wang

**Affiliations:** 1 Department of Endocrinology, Affiliated Zhongda Hospital of Southeast University, No. 87 DingJiaQiao Road, Nanjing, 210009 People’s Republic of China; 2 School of Medicine, Southeast University, Nanjing, People’s Republic of China 210009

**Keywords:** Adipsin, Homeostasis model of assessment for insulin resistance, Mild cognitive impairment, Type 2 diabetes mellitus

## Abstract

**Background:**

The adipokine adipsin contributes to insulin resistance (IR), inflammation, and obesity, which are all regarded as high-risk factors for mild cognitive impairment (MCI) in patients with type 2 diabetes mellitus. This research aimed to uncover the role of adipsin in Chinese type 2 diabetes mellitus (T2DM) population with early cognitive dysfunction and determine whether adipsin contributes to diabetic MCI caused by IR.

**Methods:**

In our study, 126 patients with T2DM were enrolled. The Montreal Cognitive Assessment (MoCA) was used to assess cognitive impairment. Demographic data and neuropsychological test results were evaluated. Plasma adipsin level was measured by enzyme-linked immunosorbent assay.

**Results:**

The MCI group (*n* = 57) presented higher plasma adipsin levels compared with the healthy controls (*p* = 0.018). After adjustment for educational attainment, and age, begative correlations were found between plasma adipsin levels and MoCA, Mini Mental State Exam, and Verbal Fluency Test scores(r = − 0.640, *p* < 0.001; r = − 0.612, p < 0.001; r = − 0.288, *p* = 0.035; respectively). Correlation analysis demonstrated that adipsin levels were significantly positively correlated with fasting C-peptide; homeostasis model of assessment for insulin resistance (HOMA-IR) (r = 0.368, p < 0.001; r = 0.494, p < 0.001; respectively). Multivariable regression analysis further indicated that high plasma adipsin level was a significant independent determinant of MCI in the Chinese population withT2DM (*p* = 0.017).

**Conclusions:**

Elevated plasma adipsin level was associated with MCI in Chinese T2DM patients. Further large-scale studies should be designed to determine whether adipsin is linked to IR-associated susceptibility to early cognitive decline in T2DM patients.

## Background

Given its prevalence, type 2 diabetes mellitus (T2DM) is expected to affect 552 million people worldwide by 2030 according to the International Diabetes Federation (IDF) [1]. With its growing chronic complications, diabetes-induced cognitive dysfunction has received considerable attention from researches [2]. Previous researches demonstrated that patients with T2DM have an increased incidence of dementia and mild cognitive impairment (MCI), a transition phase between dementia and regular aging [3, 4]. T2DM results in a 60% increase in Alzheimer’s disease (AD) risk [5]. The exact mechanisms of diabetes-induced cognitive dysfunction are multifactorial. Insulin resistance (IR), dyslipidemia, neuroinflammation, hyperphosphorylation of TAU and abnormal accumulation of amyloid-beta (Aβ) peptide were reported [6, 7]. Nevertheless, the potential etiology and pathological mechanisms remain unclear.

IR is one of the principal distinctive features of T2DM, which exists throughout the entire diabetes course [8]. IR itself also leads to the production of Aβ and hyperphosphorylation of tau protein [9]. Prior studies suggested that systemic IR actuates brain IR [9], and leads to the reduction of cerebral glucose metabolic rate and worsened memory [10]. Indeed, accumulated evidence has suggested that AD is usually accompanied by profound IR; moreover, IR abnormalities also participate in the occurrence of T2DM-related early cognitive dysfunction and contribute to the progression of MCI to AD [11]. However, the precise mechanisms about diabetic MCI caused by IR remain uncertain.

Partial adipocytokines were thought to be involved in diabetic-related MCI. Pathological mechanisms such as cerebral IR, hyperinsulinemia, and inflammation have been discussed. Certain adipocytokines including leptin and adiponectin were reported to medicate early cognitive impairment caused by IR [12, 13]. Leptin-deficient mice with T2DM [14] show impaired cerebral insulin signaling, thereby leading to the activation of glycogen synthase kinase 3β(GSK3β), the production of Aβ, the hyperphosphorylation of tau protein, and subsequent cognitive impairment. The adipocytokine adiponectin can ameliorate insulin sensitivity by activating protein kinase (AMPK) phosphorylation, reaulting in neuroinflammation, neurodegeneration, Aβ production, and tau protein hyperphosporylation. Thus, IR plays an important role in T2DM, adipokine levels, and cognitive impairment. The adipokine adipsin (complement factor D), is a serine protease that was first found in 3 T3 adipocytes [15]. Patients suffering from DM have high serum and cerebrospinal fluid (CSF) levels of adipsin [16]. In mice, Lo et al. found that adipsin, together with its downstream receptor of C3a, C3aR1, acts on islets and finally stimulates insulin secretion [17]. This finding provided a link between IR and adipsin. The association between adipsin and IR has been confirmed in some investigations. Many human clinical studies presented a positive correlation between adipsin and IR, although contradictory clinical reports were found by Wang et al. [18]. Moreover, adipsin has also been reported to modulate lipid metabolism [19], ischemia-reperfusion [20], and insulin secretion [17], which are all implicated as risk factors of cognitive dysfunction. Thus, adipsin, probably plays a previously unrecognized role in T2DM-related cognitive dysfunction. Therefore, we hypothesized that adipsin might regulate IR - related susceptibility to early cognitive dysfunction in T2DM patients.

The present cross-sectional study aimed to evaluate the latent correlation between plasma adipsin levels and diabetes-related cognitive impairment. Further analysis may reveal the potential mechanisms of IR- related susceptibility to early cognitive impairment in T2DM patients.

## Methods

### Clinical subjects and study design

The present cross-sectional research was designed and implemented in T2DM patients from 2013 to 2017. The Endocrinology Department of the Affiliated Zhongda Hospital of Southeast University provided recruiters. Altogether, 126 right-handed, hospitalized T2DM individuals were recruited (71 men and 55 women, aged 40–75 years). All subjects had at least three years of diabetes duration and met the diagnostic criteria for T2DM based on the World Health Organization in 1999 [21]. Among these individuals, 57 patients (28 females, 29 males, mean ± SE age = 59.98 ± 0.919 years) were diagnosed as MCI and 69 patients (27 females, 42 males, mean ± SE age = 58.28 ± 1.035 years) were diabetic patients with healthy cognition. The recruited individuals with MCI met the 2006 diagnostic criteria: 1) Cognitive complaints, come from patients themselves or family members; 2) Clinical Dementia Rating (CDR) score ≥ 0.5); 3) Cognitive dysfunction certified by professional clinicians without dementia and major repercussions in daily life [22]. Exclusion criteria include: 1) diabetic ketoacidosis, severe hypoglycemia coma or other acute diabetic complication, 2) acute cardiovascular and cerebrovascular events, known history stroke within one year (Hachinski score ≥ 4), epilepsy, head injury, moderate depression or other psychiatric illness; 3) Severe systemic disease (i.e., thyroid disease, serious infection and anemia); 4) Severe visual or hearing loss.

### Clinical data collection

Demographic data were gathered including age, sex, education levels, height, hip circumference, waist circumference, weight, and blood pressure. Physical data were measured by a professional research staff based on a standard and uniform method. The body mass index (BMI) = body weight in kilograms / the square of the height in meters (kg/m2). Systolic blood pressure ≥ 140 mmHg or diastolic blood pressure ≥ 90 mmHg would be defined as hypertension, according to the 2010 Chinese Hypertension Management Guidelines [23]. Medical histories such as diabetes duration (calculated from the time when diabetes was diagnosed by a professional doctor), insulin use, lifestyle factors (including smoking and drinking) were obtained through self-report or medical records. Fatty liver was detected by the Color Doppler ultrasound. The blood samples were assayed for fasting and 2-h postprandial glucose (FBG and 2hPG), glycosylated hemoglobin (HbA1c), total cholesterol (TC), fasting C-peptide (FCP), triglyceride (TG), high-density and low-density lipoprotein cholesterol (HDL-C, LDL-C), apolipoprotein A1 (ApoA1) and apolipoprotein B (ApoB). The homeostasis model of assessment for insulin resistance (HOMA-IR) was calculated by the formula HOMA-IR = fasting glucose (mmol/L) x fasting peptide (nmol/L) / 22.5 [24]. All the samples were measured by the Center of the Zhongda Hospital in accordance with the internal and external quality management procedures implemented by the Chinese Laboratory of Quality Control.

### Neuropsychological test data

An experienced neuropsychiatry specialist conducted the neuropsychological test by using a single-blind method. The present research employed a neuropsychological battery, including Montreal Cognitive Assessment (MoCA), Verbal Fluency Test (VFT), Mini Mental State Exam (MMSE), Clock Drawing Test (CDT), Digit Span Test (DST), Auditory Verbal Learning Test (AVLT), Stroop color word test (SCWT), Trail Making Test-A and B (TMT-A and TMT-B). Overall cognitive function, executive abilities, calculation ability, attention and information processing speed were covered.

### Measurement of plasma adipsin level

After overnight fasting, 2 ml blood samples were drawn from the anterior elbow vein between 6 and 7 A. M into tubes anticoagulated by heparin and then centrifuged at 100×g at least 15 min. After that, the samples were separated and refrigerated at − 80 °C before measured. The plasma levels of adipsin were detected by the enzyme-linked immunosorbent assay kits [USCN, Wuhan, China] based on the manufacturer’s instructions. The Intra-Assay CV was < 10% and the Inter-Assay CV < 12%. The minimum detectable value of this kit was 0.257 ng/ ml. Each sample was measured 2 times and then taking the average value. All samples were measured on the same day to minimize test variation.

### Statistical analysis

All the data were tested in the form of the means ± standard error (SE), n (%), or the median (interquartile range) according to the characteristics. SPSS version 22.0 was conducted. The Kolmogorov –Smirnov (KS) test was performed to validate the normality of data. Analysis of variance (ANOVA) and Student’s tests were performed for normally distributed variables, otherwise, non-parametric Mann-Whitney U or Kruskal-Wallis tests would be performed. Besides, the Chi-squared analysis (χ2) was taken to analyze categorical data. The partial correlation analysis was used after adjustment for age and some other confounding factors to determine the correlation of plasma adipsin levels and cognitive performance. The Regression model was conducted to establish a predictive model of MCI. MCI group was recommended with a MoCA score less than 26, patients with education levels < 12 years would have a one-point adjustment. All analyses were bilateral. *P* < 0.05 was considered statistically significant.

## Results

### Demographic, clinical and neuropsychological characteristics

The demographic, clinical and neuropsychological tests are shown in Table 1. A total of 126 Chinese subjects with T2DM were recruited and further divided into two groups. Among these patients, 57 were diagnosed as MCI and 69 showed healthy cognition.

**Table 1:** Demographic, clinical and cognitive performances of patients with T2DM

Characteristic	MCI group(n = 57)	Non-MCI group (*n* = 69)	*p*-value
Age (years)	59.98 ± 0.919	58.28 ± 1.035	0.229^a^
Female, n(%)	28 (49.1%)	27 (39.1%)	0.26^c^
Education Levels (years)	10 (9–12)	11 (9–12)	0.619^b^
Smoking, n(%)	18 (31.6%)	25 (36.2%)	0.632^c^
Drinking, n(%)	11 (19.3%)	18 (26.1%)	0.368^c^
Hypertension, n (%)	36 (63.2%)	39 (56.5%)	0.45^c^
SBP (mmHg)	134.81 ± 18.55	135.29 ± 15.79	0.875^a^
DBP (mmHg)	81.30 ± 11.33	80.10 ± 9.76	0.525^a^
Hypertension duration (years)	5 (0–12)	3 (0–12)	0.440^b^
Diabetes duration (years)	10.807 ± 0.686	9.529 ± 0.655	0.182^a^
Insulin use, n(%)	33 (57.9%)	42 (60.9%)	0.735^c^
metformin, n(%)	34 (59.6%)	39 (56.5%)	0.432
Glucose fluctuation (mmol/L)	6.55 ± 0.38	6.71 ± 0.36	0.766^a^
HbA1c (%)	9.72 ± 0.35	8.79 ± 0.25	0.028^a^
FBG (mmol/L)	8.599 ± 0.34	7.662 ± 0.30	0.041^a^
@2hPG (mmol/L)	15.15 ± 0.49	14.37 ± 0.48	0.262^a^
FCP (ug/L)	1.25 (0.63–1.92)	0.779 (0.44–1.4)	0.025^b*^
HOMA-IR	0.434 (0.227–0.665)	0.251 (0.146–0.472)	0.005 ^b*^
BMI (kg/m^2^)	24.94 ± 0.44	24.73 ± 0.37	0.723^a^
Weight (kg)	68.58 ± 1.39	69.60 ± 1.49	0.619^a^
WC (cm)	90.11 ± 1.14	88.38 ± 1.22	0.310^a^
HC (cm)	95.61 ± 0.78	94.39 ± 7.25	0.307^a^
WHR	0.942 ± 0.008	0.937 ± 0.008	0.858^a^
TG (mmol/L)	1.80 ± 0.85	1.73 ± 0.13	0.72^a^
TC (mmol/L)	4.77 ± 0.14	4.56 ± 0.13	0.297^a^
HDL (mmol/L)	1.19 ± 0.05	1.17 ± 0.03	0.889^a^
LDL (mmol/L)	2.97 ± 0.113	2.82 ± 0.097	0.328^a^
ApoA1(g/L)	1.09 ± 0.035	1.08 ± 0.030	0.759^a^
ApoB(g/L)	0.84 ± 0.029	0.815 ± 0.022	0.663^a^
Fatty liver, n(%)	29 (50.9%)	28 (40.6%)	0.248^c^
Adipsin (μg/ml)	13.532 ± 0.948	10.4274 ± 0.877	0.018^a*^
Neuropsychological test scores			
MOCA	23 (20–24)	27 (27–28)	< 0.001^b**^
MMSE	26 (23–28)	29 (28–30)	< 0.001^b**^
CDT	3 (2–4)	4 (3–4)	0.032^b*^
DST	10.49 ± 0.27	10.49 ± 0.221	< 0.001^a**^
VFT	14.25 ± 0.421	16.59 ± 0.425	< 0.001^a**^
TMTA	66 (53–84)	52 (45–57)	< 0.001^b**^
TMTB	198.28 ± 11.881	139.96 ± 5.737	< 0.001^a**^
SCWT A(time)	33 (28–38)	28 (24–35)	0.013^b*^
SCWT A (number)	50 (50–50)	50 (50–50)	0.03^b*^
SCWT B(time)	57 (46–62)	43 (36–49)	< 0.001^b*^
SCWT B (number)	48 (46–50)	50 (49–50)	< 0.001^b**^
SCWT C(time)	109 (82–123)	82 (71–89)	< 0.001^b**^
SCWT C (number)	44 (42–47)	48 (46–50)	< 0.001^b**^
AVLT immediate	15.74 ± 0.698	18.93 ± 0.456	< 0.001^a**^
AVLT delayed	5 (3–6)	6 (5–7)	< 0.001^b**^
LMT	6.16 ± 0.558	10.12 ± 0.537	< 0.001^a**^

Significance, *p<0.05; **p < 0.01

Data are presented as n (%), mean ± SE, or median (interquartile range) as appropriate

^a^ Student’s t test for comparison of normally distributed quantitative variables between MCI group and N-MCI group

^b^ Mann-Whitney U test for comparison of asymmetrically distributed quantitative variables between MCI group and N-MCI group

^c^ χ2 test for comparison of qualitative variables between MCI group and N-MCI group

Abbreviations: MCI, mild cognitive impairment; SBP, systolic blood pressure; DBP, Diastolic blood pressure; HbA1c, glycosylated hemoglobin; FBG, fasting blood-glucose; 2hPG, 2-h postprandial blood glucose; FCP, fasting C-peptide; HOMA-IR, homeostasis model of assessment for insulin resistance; BMI, body mass index; WC, waist circumference;HC, hip circumference; TG, triglyceride; TC, total cholesterol; HDL, high-density lipoprotein; LDL, low-density lipoprotein; ApoA1, apolipoprotein A1; ApoB, apolipoprotein B; MoCA, Montreal Cognitive Assessment; MMSE, Mini-mental State Examination; CDT, Clock Drawing Test; DST, Digit Span Test; VFT, Verbal Fluency Test; TMT-A, Trail Making Test-A; TMT-B, Trail Making Test-B; SCWT, Stroop Color Word Test; AVLT, Auditory Verbal Learning Test; LMT, Logical Memory Test

The two groups well matched in terms of age, sex, educational attainment, smoking, drinking, hypertension, insulin use and duration of diabetes (all *p* > 0.05). No significant differences were discovered in both groups in BMI, weight, WC, HC, WHR, TG, TC, HDL, LDL, ApoA1 and ApoB (all p > 0.05). Compared with the normal group, the MCI group exhibited increased plasma FCP, FBG, HOMA-IR and HbA1c levels (all *p* < 0.05). Moreover, increased adipsin levels were found in the MCI group (13.532 ± 0.948 vs. 10.4274 ± 0.877, p < 0.05). T2DM patients with MCI presented poorer cognitive performance than healthy controls (all p < 0.05).

### Relationship between plasma adipsin level and cognitive performance

The correlation between the plasma levels of adipsin and cognitive performance were determined by partial correlation analysis for all subjects. After adjusting for age, sex and education levels, the adipsin level was statistically significant negatively correlated with MoCA scores (r = − 0.640, *p* < 0.001), MMSE scores (r = − 0.612, p < 0.001), SCWT-A Time(r = 0.290, *p* = 0.034) and VFT scores (r = − 0.288, *p* = 0.035) in T2DM patients with cognition dysfunction. However, only the SCWT-A Number(r = − 0.299, *p* = 0.015) was interrelated in the normal cognitive group (Table 2).

**Table 2:** Correlation analysis of the plasma adipsin level and the neuropsychological test results in different groups

	MCI group		Non-MCI group	
	r	p	r	p
MoCA	−0.640	< 0.001	− 0.057	0.648
MMSE	−0.612	< 0.001	− 0.187	0.132
SCWT-A Time	0.290	0.034	−0.114	0.365
SCWT-A Number	−0.202	0.142	−0.299	0.015
SCWT-B Time	0.069	0.618	−0.193	0.121
SCWT- B Number	−0.008	0.953	0.012	0.921
SCWT-C Time	0.156	0.260	−0.003	0.978
SCWT-C Numbe	0.153	0.268	0.026	0.834
AVLT immediate recall	−0.154	0.266	−0.045	0.720
AVLT delayed recall	−0.082	0.554	0.014	0.911
CDT	−0.077	0.579	−0.128	0.304
LMT	0.050	0.722	−0.076	0.542
DST	−0.218	0.114	0.056	0.655
VFT	−0.288	0.035	−0.101	0.422
TMTA	0.062	0.658	0.032	0.797
TMTB	0.176	0.204	0.200	0.107

Abbreviations: MCI, mild cognitive impairment; MoCA, Montreal Cognitive Assessment; MMSE, Mini-mental State Examination; CDT, Clock Drawing Test; DST, Digit Span Test; VFT, Verbal Fluency Test; TMT-A, Trail Making Test-A; TMT-B, Trail Making Test-B; SCWT, Stroop Color Word Test; AVLT, Auditory Verbal Learning Test; LMT, Logical Memory Test

### Binary logistic regression analysis for all individuals

We elucidated the major determinants related to MCI prevalence in the enrolled populations. A regression analysis was constructed. All variables from Table 1 were entered into the model. The model was finallydeveloped by a stepwise approach. Adipsin (β = 0.063, *p* = 0.017) and HbA1c (β = 0.196, *p* = 0.031) were eventually imported to the model (Table 3), and analysis revealed that high plasma levels of adipsin and HbA1c were the independent risk factors that increased the diagnosis of MCI.

**Table 3:** Assessment results of the risk of having MCI in a binary regression model in patients with T2DM

	β	SE	95% CI		p
			Lower	Upper	
adipsin	0.063	0.026	1.012	1.121	0.017
HbA1c	0.196	0.091	1.018	1.454	0.031

Abbreviations: T2DM, type 2 diabetes mellitus; β, regression coefficient; SE, standard error; CI, confidence interval for odds ratio; HbA1c, glycosylated hemoglobin

### Multivariable regression analysis among patients with cognitive impairment

Simple linear regression models and multivariable linear regression models were constructed to evaluate the independent factors that might affect the neuropsychological test results. The MoCA score was taken as a dependent variable, while sex, age, education level, diabetes duration, HbA1c, FBG, and adipsin were included as independent variables in the multiple stepwise regression models. The analysis results suggested that MoCA score was negatively associated with adipsin, (standardized B = -0.623, *p* < 0.001; β = − 0.286, *p* = 0.02; respectively; Table 4). Similar results were obtained with MMSE and VFT as the dependent variables (Table 4).

**Table 4:** Multivariable linear regression analyses of clinical indicators and MoCA, MMSE, VFT scores in the MCI subgroup

	standardized B	adjusted R^2^	SE	95% CI		p
				Lower	Upper	
MoCA						
adipsin	−0.623	0.38	0.044	−0.349	− 0.172	< 0.001
MMSE						
adipsin	−0.601	0.35	0.048	−0.364	−0.172	< 0.001
VFT						
adipsin	−0.259	0.064	0.056	−0.226	−0.002	0.045
HbA1c	−0.257		0.152	−0.613	−0.005	0.047

Abbreviations: B, regression coefficient; SE, standard error; CI, confidence interval for odds ratio; HbA1c, glycosylated hemoglobin; MoCA, Montreal Cognitive Assessment; MMSE, Mini-mental State Examination; VFT, Verbal Fluency Test

### Correlations of adipsin with clinical variables

Table 5 shows the correlations between the plasma adipsin level and different clinical data. Spearman rank correlation or Pearson correlation analyses were performed. Remarkable positive correlations were found in FCP (r = 0.368, *p* = 0.005; r = 0.525, p < 0.001) and HOMA-IR (r = 0.494, p < 0.001, r = 0.437;p < 0.001) in all participants. In addition, positive correlations were found between plasma adipsin level and SBP (r = 0.285, *p* = 0.032), hypertension (r = 0.463, p < 0.001), smoking (r = 0.317, *p* = 0.016), BMI(r = 0.336, *p* = 0.011), and weight (r = 0.295, *p* = 0.029) in the MCI group only. No statistically significant differences were found between adipsin and WC and adipsin and fatty liver.

**Table 5:** Association between plasma level of adipsin and clinical variables

	MCI group		Non-MCI group	
	r	p	r	p
SBP	0.285	0.032	0.009	0.943
FCP	0.368	< 0.001	0.525	< 0.001
Hypertension	0.463	< 0.001	0.235	0.052
Smoking	0.317	0.016	0.067	0.587
Fatty liver	−0.166	0.216	−0.079	0.521
HOMA-IR	0.494	< 0.001	0.437	< 0.001
BMI	0.336	0.011	0.035	0.778
2hPG	−0.017	0.898	−0.293	0.015
APoB	−0.038	0.781	−0.292	0.015
FBG	−0.006	0.967	−0.2	0.1
WC	0.213	0.112	0.089	0.469
TC	0.135	0.317	−0.109	0.374
TG	0.024	0.857	−0.01	0.936

Abbreviations: MCI, mild cognitive impairment; SBP, systolic blood pressure; FCP, fasting C-peptide; HOMA-IR, homeostasis model of assessment for insulin resistance; BMI, body mass index; 2hPG, 2-h postprandial blood glucose; ApoB, apolipoprotein B; FBG, fasting blood-glucose; WC, waist circumference;TC, total cholesterol; TG, triglyceride

### Multiple linear regression analysis

Multiple linear regression analyses were performed to evaluate the independent associations between adipsin and clinical parameters (Table 6). All the independent parameters were entered in the model at step one. APoB, FCP, HOMA-IR, TC, and FBG independently predicted adipsin levels.

**Table 6:** Evaluation of the effects of clinical indicators on adipsin in T2DM by multiple linear regression analysis

Variables analyzed	β	SE of β	95% CI		p
			Lower	Upper	
TC	1.761	0.801	0.174	3.348	0.030
FBG	−0.642	0.285	−1.207	−0.078	0.026
HOMA-IR	12.854	3.061	6.793	18.915	< 0.001
FCP	−1.576	0.721	−3.004	−0.149	0.031
TG	−0.522	0.545	−1.601	0.557	0.340

Abbreviations: T2DM, type 2 diabetes mellitus; β, regression coefficient; SE, standard error; CI, confidence interval for odds ratio; TC, total cholesterol; FBG, fasting blood-glucose; HOMA-IR, homeostasis model of assessment for insulin resistance; FCP, fasting C-peptide; TG, triglyceride

## Discussion

The foremost results of this study were as follows: (1) Compared with the normal controls, individuals with cognitive dysfunction exhibited higher plasma adipsin levels. (2) After controlling for potential confounders such as levels of education, age, and sex, the plasma adipsin level was remarkably negatively correlated with MoCA, MMSE and VFT scores, which represent executive function [25] in the MCI group. (3) Increased plasma adipsin level was a major independent determinant for diabetic MCI. (4) Plasma adipsin level was positively associated with FCP and HOMA-IR in all subjects. In addition, FCP and HOMA-IR independently predicted adipsin levels.

Consistent with previous studies [26], our study demonstrated that T2DM patients with MCI exhibited worsened glucose homeostasis, as indicated by increased HbA1c and FBG in the MCI group. Moreover, HbA1c is an independent risk factor for poor cognitive performance. Yaffe et al. [27] reported that for patients with HbA1c ≥ 7%, the risk for developing MCI was increased by nearly fourfold in a large sample size study with a female population. Several mechanisms have been investigated, including accumulation of advanced glycation end-products, activation of protein kinase C and increased flux of hexosamine in brain endothelial cells that lead to vessel occlusion, alteration of angiogenesis and permeability, production of NF-κB that causes neuroinflammation, obstruction of Akt/CREB signaling pathway, and impaired insulin homeostasis of the brain [26, 28].

Moreover, increased plasma adipsin level was correlated with higher BMI and weight in the MCI group. Adipose tissue dysfunction and obesity exists in diabetes and contributes to the development of lipid metabolism disorder and MCI in patients with T2DM [29–31]. Animal experiments indicated that circulating adipsin levels decreased in obese models [32], while human studies present diametrically opposite results [33]. The exact explanation for these discrepancies remains uncertain. One possible reason is that the expansion of fat mass in obesity may compensate to maintain higher circulating levels of adipsin [17]. Our results were consistent with most human studies. Maslowska et al. [34] reported that an obese group presented a high plasma adipsin levels, and a strong positive association was discovered between BMI and plasma adipsin. Analogous results were found in a cross-sectional study carried out on Arabs [35],in which a positive correlation was also discovered between adipsin and waist circumference. Schrover et al. [36] reported a strong positive relation between adipsin and BMI; moreover, visceral adipose tissue was related to higher plasma concentrations of adipsin. Conversely, adipsin is a serine protein in triglyceride synthesis through the ASP/adipsin pathway [19, 37]. Maslowska et al. [34] also verified that free fatty acidand BMI predicted adipsin levels. However, insignificant correlations were found between adipsin and TG and adipsin and WC in our study.

We also noted significantly higher FCP and HOMA-IR in the MCI group, thereby suggesting an association between elevated IR and MCI. Our findings are in line with previous research supporting the concept that IR contributes to the pathological mechanism of cognitive dysfunction [38]. Consistent with our study, Ekblad et al. [39] reported that IR predicted poor verbal fluency and acted as an independent risk factor of mild cognitive dysfunction in a population-based cohortwith an 11-year large follow-up survey. Insulin signaling in the brain induces the suppression ofGSK-3, which results in tau hyperphosphorylation and neurofibrillary formation, thereby causing damage or apoptosis of neurons [9].

Our correlation study of plasma adipsin level and IRrevealed that higher plasma adipsin level was correlated with higher FCP and HOMA-IR. Further multivariate analysis confirmed that the degree of HOMA-IR was an independent predictor of adipsin levels. Research on animals suggested that adipsin cleaves factor B, thereby catalyzing the formation of C3bBb, which cleaves C3 to liberate C3a. Then, together with its downstream receptor of C3a, C3aR1 acts on islets through augmenting intracellular adenosine-triphosphate (ATP) levels, thus motivating ATP-coupled respirations, increasing the concentration of cytosolic free Ca2+, and finally stimulating insulin secretion [17]. Interestingly, a dispute exists about the relationship between adipsin and IR. Our findings are consistent with some human studies. One recent cross-sectional study displayed significant positive correlations between adipsin levels and the HOMA-IR index in patients with polycystic ovary syndrome. In another study, IR was found to be an independent predictive factor of adipsin levels [40]. Similar findings were obtained by Derosa et al. [41], who suggested that increased HOMA-IR index was profoundly correlated with higher adipsin levels in obese subjects. However, an insignificant correlation was reported in a study on t Arabs with cardiovascular disease. Lo et al. [17] revealed that adipsin−/− mice are insulinopenic, and diabetic db/db mice injected with adipsin presented increased insulin secretion and improved glucose homeostasis. Wang et al. reported that serum adipsin levels were negatively correlated with IR, especially in subjects with BMI ≥ 25 kg/m^2^. The disparity between results was not fully understood. One possible explanation is that the patients with T2DM employed in our research have not developed severe metabolic disorders, and a compensatory mechanism may have been triggered to retain normal insulin secretion. In addition, different study populations (patients with MCI in our report vs. subjects with cardiovascular disease by Calan et al. [41]), different races (Asians vs. Arabs), and different species (human vs. mice) may at least partly explain such discrepancies. Additionally, MCI subjects presented high adipsin levels. Further multivariate analyses demonstrated that, besides HbA1c, adipsin is an independent determinant for MCI individuals with T2DM. This outcome implies that a higher plasma adipsin level might play a previously unrecognized role in diabetic MCI caused by IR. Neuropsychological tests conducted in our study also indicated that the MMSE and MoCA scores, which represented global cognition, were inversely correlated to plasma adipsin levels. The influences on cognitive dysfunction were consistent with cognitive decline caused by insulin resistance in the past study conducted by Zhong Y et al. [42]. In our present study, although lower HOMA-IR was found in the MCI group, the further multivariable linear regression analyses discovered insignificant correlations about HOMA-IR and MoCA, MMSE, VFT scores. This may be due to the insufficient population. Furthermore, adipsin acts as an independent predictor of VFT, which represents the executive function. To the best of our knowledge, this is the unique research estimating the expressing of adipsin in cognitive impairment with T2DM.

One or more possible explanations may be reasonable for the observed results: 1) glucose dysregulation in T2DM patients with cognitive dysfunction promotes adipsin production as a compensatory mechanism to maintain normal insulin secretion [17], that leads to compensatory IR, which, in turn may contribute to impairment of cellular insulin signaling [43], reduction of brain insulin uptake, and increased levels of Aβ [10]. In addition, abnormal insulin signaling represents a risk to the dysfunction of the entorhinal cortex and hippocampus, finally leading to impaired memory and executive function [44]. 2) adipsin itself is related to CSF inflammation and increases grades of disturbed blood-CSF barrier function [16].

Several limitations exist in our research. First, the cross-sectional study design itself failed to explain any causal relationship between adipsin and cognitive impairment. Large-scale and multi-center prospective studies should be conducted to verify our inferences. Second, some clinical parameters such as education levels were collected through self-report and medical records, which could lead to recall bias. Third, the sample size in this study is not large enough and consists of the Chinese Han population, which might reduce the strength of the results. Furthermore, while we adjusted some confounding factors that may influence the logistic regression, the results may have been affected by other possible confounders (e.g., living environment and habits), which could not be controlled. Finally, we used the less invasive HOMA-IR method to evaluate IR; thus, more accurate indicators should be used to gain a better evaluation of IR.

## Conclusion

In summary, the current study demonstrated that T2DM individuals with cognitive dysfunction presented increased plasma adipsin levels. Furthermore, high plasma adipsin level is an independent risk predictor of overall cognitive function and executive function. The data implied that adipsin could be a potential predictor of early cognitive dysfunction among Chinese patients with T2DM. Additionally, we obtained evidence that plasma adipsin level is significantly positively associated with FCP and HOMA-IR, which suggested that adipsin might facilitate the development of diabetic MCI caused by IR. Further prospective studies with large sample size should be conducted to confirmed our hypotheses and clarify whether adipsin is involved in diabetic MCI caused by IR.

## Data Availability

The datasets used and/or analyzed during the current study are available from the corresponding author on reasonable request.

## Abbreviations

2hPG: 2-h postprandial blood glucose
AD: Alzheimer’s disease
ApoA1: apolipoprotein A1
ApoB: apolipoprotein B
AVLT: Auditory Verbal Learning Test
BMI: body mass index
CDT: Clock Drawing Test
CI: confidence interval for odds ratio
DBP: Diastolic blood pressure
DST: Digit Span Test
FBG: fasting blood-glucose
FCP: fasting C-peptide
HbA1c: glycosylated hemoglobin
HC: hip circumference
HDL: high-density lipoprotein
HOMA-IR: homeostasis model of assessment for insulin resistance
IR: insulin resistance
LDL: low-density lipoprotein
LMT: Logical Memory Test
MCI: mild cognitive impairment
MMSE: Mini-mental State Examination
MoCA: Montreal Cognitive Assessment
SBP: systolic blood pressure
SCWT: Stroop Color Word Test
SE: standard error
T2DM: type 2 diabetes mellitus
TC: total cholesterol
TG: triglyceride
TMT-A: Trail Making Test-A
TMT-B: Trail Making Test-B
VFT: Verbal Fluency Test
WC: waist circumference
β: regression coefficient
